# P59. Depleting the suppressors for the benefit of immunotherapy against cervical cancer

**DOI:** 10.1186/2051-1426-2-S2-P33

**Published:** 2014-03-12

**Authors:** O Draghiciu, BN Hoogeboom, T Meijerhof, HW Nijman, CAHH Daemen

**Affiliations:** 1 University Medical Center Groningen, Medical Microbiology, Groningen, the Netherlands; 2 University Medical Center Groningen, Gynaecology, Groningen, the Netherlands

## Background

Cancer vaccines aim at inducing tumour-specific immune responses. However, in clinical studies so far these approaches have limited antitumour effect. Evidence is accumulating that MDSC (myeloid-derived suppressor cells) can suppress the antitumour immune response. Reversal of MDSC-mediated immune suppression by treatment with the tyrosine kinase inhibitor sunitinb can therefore possibly increase the efficacy of cancer vaccines.

## Material and methods

We developed a method to assess MDSC depletion in a preclinical model of HPV-induced neoplasia. TC-1 (cells expressing HPV16-E7) tumour-bearing mice were injected with different dosages of sunitinib daily, for 9 days, with and without immunisation with SFVeE6,7 (Semliki Forest virus encoding human papilloma virus E6,7 tumor antigens). Intra-tumoral, intra-splenic and circulating MDSC and CD8 T cell levels were assessed after treatment.

## Results

Upon sunitinib treatment, the absolute numbers of intra-tumoral, intra-splenic and circulating MDSC decreased in a dose-dependent manner. Combined sunitinib and immunisation therapy led to a marked decrease of intra-tumoral, intra-splenic and circulating MDSC levels as compared to non-treated control or immunisation alone. The bi-therapy regimen markedly enhanced intra-tumoral, intra-splenic and circulating levels of CD8 T cells. The highest number of circulating CD8 T cells undergoing degranulation (CD107ab+) was observed after combined treatment. Most importantly, this combined sunitinib and immunisation treatment regimen abrogated tumour growth.

## Conclusions

In summary, we demonstrated that sunitinib alone or in combination with immunisation can decrease intra-tumoral, intra-splenic and circulating MDSC levels. Also, combination of sunitinib treatment with immunisation enhanced levels and degranulating activity of CD8 T cells, thus resulting in reversal of tumour growth. This study indicates that SFV-based immunotherapy combined with sunitinib treatment could improve treatment outcome.

